# Analysis and Prediction of Translation Rate Based on Sequence and Functional Features of the mRNA

**DOI:** 10.1371/journal.pone.0016036

**Published:** 2011-01-06

**Authors:** Tao Huang, Sibao Wan, Zhongping Xu, Yufang Zheng, Kai-Yan Feng, Hai-Peng Li, Xiangyin Kong, Yu-Dong Cai

**Affiliations:** 1 Key Laboratory of Systems Biology, Shanghai Institutes for Biological Sciences, Chinese Academy of Sciences, Shanghai, People's Republic of China; 2 Shanghai Center for Bioinformation Technology, Shanghai, People's Republic of China; 3 Shanghai Key Laboratory of Bio-Energy Crops, School of Life Sciences, Shanghai University, Shanghai, People's Republic of China; 4 Institute of Health Sciences, Shanghai Institutes for Biological Sciences, Chinese Academy of Sciences and Shanghai Jiao Tong University School of Medicine, Shanghai, People's Republic of China; 5 Department of Physiology and Biophysics, School of Life Sciences, Fudan University, Shanghai, People's Republic of China; 6 CAS-MPG Partner Institute for Computational Biology, Shanghai Institutes for Biological Sciences, Chinese Academy of Sciences, Shanghai, People's Republic of China; 7 State Key Laboratory of Medical Genomics, Ruijin Hospital, Shanghai Jiaotong University, Shanghai, People's Republic of China; 8 Institute of Systems Biology, Shanghai University, Shanghai, People's Republic of China; 9 Centre for Computational Systems Biology, Fudan University, Shanghai, People's Republic of China; University of Edinburgh, United Kingdom

## Abstract

Protein concentrations depend not only on the mRNA level, but also on the translation rate and the degradation rate. Prediction of mRNA's translation rate would provide valuable information for in-depth understanding of the translation mechanism and dynamic proteome. In this study, we developed a new computational model to predict the translation rate, featured by (1) integrating various sequence-derived and functional features, (2) applying the maximum relevance & minimum redundancy method and incremental feature selection to select features to optimize the prediction model, and (3) being able to predict the translation rate of RNA into high or low translation rate category. The prediction accuracies under rich and starvation condition were 68.8% and 70.0%, respectively, evaluated by jackknife cross-validation. It was found that the following features were correlated with translation rate: codon usage frequency, some gene ontology enrichment scores, number of RNA binding proteins known to bind its mRNA product, coding sequence length, protein abundance and 5′UTR free energy. These findings might provide useful information for understanding the mechanisms of translation and dynamic proteome. Our translation rate prediction model might become a high throughput tool for annotating the translation rate of mRNAs in large-scale.

## Introduction

It is often assumed that genes with high mRNA levels also have high protein abundance. Thus, mRNA levels are used instead of protein abundance. However, the regulation of gene expression takes place at many levels, from transcription to translation and to the post-translational modification. Many studies either could not find the assumed correlation between mRNA level and protein abundance [1] or the correlation was very weak[2], [3]. By estimation, only 20%–40% of protein abundance is determined by the concentration of its corresponding mRNA [4], [5]. The reason for such weak correlation between protein and mRNA levels is that protein concentrations depend not only on the mRNA level, but also the translation rate and the degradation rate [6].

Translation is the third process of gene expression. In this stage, mRNA is decoded by the ribosome which binds to tRNAs with complementary anticodon sequences. The tRNAs carry specific amino acids that are synthesized into a polypeptide as the mRNA passes through the ribosome. Translation has three steps: initiation, elongation and termination [7]. Both empirical and theoretical studies showed that the bottleneck step in the translation process is the initiation of protein translation [8], [9], [10]. At the initiation step, the ribosome binds to the five prime untranslated region (5′UTR) of mRNA and moves along the mRNA until it gets to the translation start site (TSS). After initiation is completed, the ribosome enters the elongation stage. At elongation step, the ribosome waits until it intercepts an appropriate tRNA whose anticodon complements the codon at the A site of ribosome. Once the correct tRNA is intercepted by the ribosome, the amino acid from the tRNA is transferred to the ribosome associated peptide chain, and the ribosome moves forward one codon. The waiting for the correct tRNA limits the elongation process [10], [11]. Translational initiation rate determines protein production rate and elongation rate determines ribosome occupancy [8]. Therefore, ribosome density is proportional to translational initiation rate which determines protein production while it is inversely proportional to translational elongation rate.

The regulation of translation plays as important role as transcriptional regulation in the control of gene expression. Changes of the mRNAs translation rate have great influence on the actual protein abundance. Dysregulation of translation will result in various diseases, such as cancer and neurological disorders [12].

With ribosome-profiling technology, ribosome-protected mRNA fragments can be deep-sequenced and the translation rate can be monitored, but it is time-consuming, expensive and not helpful for understanding the translation mechanisms. Here we choose *Saccharomyces cerevisiae*, one of the most studied model organisms, to perform our study and predict the translation rate. We used the ribosome-profiling data from Ingolia's work [13] in which the read density of mRNA is measured by deep sequencing of ribosome-protected mRNA fragments under both rich and starvation conditions. According to Ingolia's work [13], the translation rate (or called as translation efficiency) is defined as the normalized read density of translation (footprints) divided by the normalized read density of transcription (mRNA). The ratio of ribosome footprints to mRNA fragments can roughly quantify the rate of protein synthesis [13] although further improvements could incorporate variations in the speed of elongation along the mRNA. Each mRNA is represented by various sequence-derived and functional features related to translation, such as codon usage frequencies, gene ontology enrichment scores, biochemical and physicochemical features, start codon features, coding sequence length, minimum free energy, 5′UTR length, 3′UTR length, number of transcription factors known to bind at the promoter region, number of RNA binding proteins known to bind its mRNA product, protein abundance, mRNA half life, protein half life and 5′UTR free energy. With this dataset, an efficient computational model to predict the translation rate was constructed with Nearest Neighbor Algorithm (NNA) and cross-validated. The prediction accuracies of jackknife cross-validation under rich and starvation condition were 68.8% and 70.0%, respectively. More specifically, to identify the most important features regulating translation rates under different conditions, we applied maximum relevance & minimum redundancy and incremental feature selection to select the important features for predicting the translation rate in rich and starvation conditions, respectively. Our results suggest that the following features are correlated with translation rate: codon usage frequency, some gene ontology enrichment scores, biochemical and physicochemical features of protein (such as amino acids composition, polarity, normalized Van Der Waals volume), number of RNA binding proteins known to bind its mRNA product, coding sequence length, protein abundance and 5′UTR free energy. Our findings might provide clues for understanding the mechanisms of translation. The translation rate prediction model could become a useful tool for annotating the translation rate of mRNAs in large-scale.

## Materials and Methods

### Dataset

The ribosome-profiling data we used are from Ingolia's work [13] and publicly available at GEOs http://www.ncbi.nlm.nih.gov/geo/query/acc.cgi?acc=GSE13750. With ribosome-profiling technology, Ingolia et al. [13] deep-sequenced the ribosome-protected mRNA fragments and monitored the genome-wide translation with subcodon resolution in *Saccharomyces cerevisiae* under both rich and starvation conditions. To get the translation rate, we divided the normalized read density of translation (footprints) by the normalized read density of transcription (mRNA) [13]. The ratio of ribosome footprints to mRNA fragments represents the translation rate and according to their values [13], we characterize the translation rates into two groups which are: (1) smaller than median or equal to median, (2) greater than median. Open Reading Frames (ORFs) in the former group have low translation rate, while the ORFs in the latter group have high translation rate. We characterized the translation rates in rich condition and starvation condition, respectively. The number of ORFs with low translation rates and high translation rates in rich condition and starvation condition can found in **Table 1**.

**Table 1:** The number of ORFs with low translation rates and high translation rates in rich condition and starvation condition.

		Starvation condition		The number of ORFs
		The number of ORFs with Low translation rate	The number of ORFs with High translation rate	
Rich condition	The number of ORFs with Low translation rate	1125	209	1334
	The number of ORFs with High translation rate	209	1124	1333
The number of ORFs		1334	1333	2667

### Feature Construction

#### Codon usage frequency features

We downloaded the ORF coding sequences from Saccharomyces Genome Database (SGD) [14] and calculated the codon relative frequencies with seqinR [15]. It was reported that highly expressed genes have different codon preference with low expressed gene and the pattern of codon usage can be used to predict the gene expression level in yeast [16]. It is highly possible that ORFs with different translation rate have different codon usage pattern, too. There were 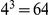 codon usage frequency features.

#### Gene Ontology features

Proteins are produced to achieve their biological functions. As demand determines production, the translation rate of ORF is definitely correlated with its biological functions. The function of one protein can be better described in protein interaction network, i.e. the network context will give a comprehensive and robust description of its function. In this study, the network context we used was STRING[17]. The Gene Ontology enrichment score of protein 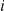 on Gene Ontology term 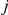
was defined as the –log_10_ of the hypergeometric test p value [18] of its neighbors on STRING network and can be computed by equation (1):
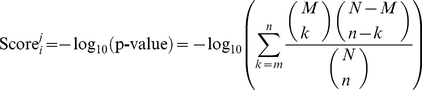
(1)where 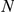 is the number of overall ORFs in yeast, 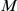 is the number of ORFs annotated to Gene Ontology term 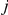, 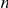 is the number of ORFs in ORF set 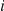 which includes protein 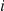
and its neighbors on STRING network, 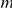 is the number of ORFs from ORF set 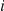 that are annotated to Gene Ontology term 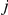. The larger the enrichment score of one Gene Ontology term, the more overrepresented this term is. There were 4148 Gene Ontology (GO) enrichment score features.

#### Biochemical and physicochemical features of proteins

To encode proteins of different sequence lengths with a uniform dimensional vector, we adopted the notion of pseudo amino acid composition (PseAAC) [19], [20]. Each protein sequence was represented by 132 biochemical and physicochemical features which can be categorized into seven groups: (1) amino acid composition [21], [22], (2) solvent accessibility, (3) normalized van der Waals volume, (4) polarizability, (5) secondary structure, (6) hydrophobicity, and (7) polarity [23]. Except for amino acid composition, all the other six ones are generated by integrating the pseudo properties of amino acid in the protein sequence and each amino acid can be classified into two or three pseudo groups. For secondary structure, each amino acid can be predicted by SSpro [24] as: helix, strand or coil. For solvent accessibility, each amino acid is predicted by ACCpro [25] as: exposed or buried to solvent. In terms of hydrophobicity, there are three groups of amino acid: hydrophobic (C, V, F, L, I, M,W), neutral (G, P, H, A, S, T,Y) and polar (Q, E, R, K, D, N)[26]. For polarizability: {Y, M, K, R,H, F,W}, {C, Q, I, P, N, V, E L} and {S, D, G, A, T} [27]. For normalized van der Waals volume: {K, F, M, H, R, Y, W}, {E, Q, N, V, I, L} and {S, C, G, A,T, P, D} [28]. For polarity: {K, N, H, Q, R,E, D}, {T, G, P, A, S} and {W, C, L, I, F,M, V, Y} [29].

To generate the global protein features by integrating the local quantities of amino acid over the entire protein sequence, the following three quantities are calculated: 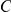
(composition), 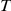
(transition), and 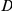
(distribution). The detailed computational procedures and a well illustrated example can be found in our previous work [30]. Generally speaking, 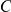
refers to the percent of each pseudo group in the sequence; 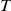
to the frequencies with which one pseudo group changes to another; and 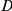
to the relative position where the first, twenty-five percent, fifty-percent, seventy-five percent, and last of each kind of pseudo letters occur.

For polarity, secondary structure, polarizability, hydrophobicity and normalized van der Waals volume, each amino acid has three pseudo groups and would generate 21 protein features. For solvent accessibility, each amino acid has two pseudo groups and would only generate 7 protein features.

Now for the amino acid composition we have 20 features; for solvent accessibility, 7 features; and for the other five properties, each has 21 features. Combining them together, each protein has 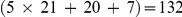 features. The detailed explanation of each biochemical and physicochemical feature can be found in our previous work [30].

#### Start codon features

During the translation initiation, the 40S subunit of ribosome binds to a site upstream of start codon. It proceeds downstream until it encounters the start codon and form the initiation complex of translation. The start codon is typically AUG (or ATG in DNA) and related with translation initiation. We extracted sequences in untranslated region 3 bp upstream of the initial ATG and sequences in coding region 3 bp downstream of the initial ATG. We encoded the 6 bp DNA sequences up/downstream of start codon ATG binarily and each base pair was represented by a 4-dementional vector: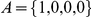,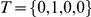, 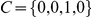
and 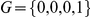.

#### Coding sequence length

We calculated the coding sequence length of each ORF as a potential feature for translation rate prediction.

#### Free energy of 42 nucleotide cross translation start site

Kudla et al. [31] identified a region, from nucleotide (nt) –4 to +37 relative to translation start site, for which predicted folding energy can explain some of the of the variation to differences in protein levels. So we calculated the minimum free energy of 42 nucleotide (nt) –4 to +37 relative to translation start site, with Vienna [32].

#### Various parameters of untranslated regions from Tuller's study

Tuller et al.[33] collected various properties of untranslated regions of the S. cerevisiae genome and we used the following 8 features from Tuller's study: 5′UTR length, 3′UTR length, Number of transcription factors known to bind at the promoter region, Number of RNA binding proteins known to bind its mRNA product, Protein abundance, mRNA half life [34], Protein half life and 5′UTR free energy[35]. Unlike the above free energy, here the 5′UTR free energy is calculated with 5′-UTR 100 nt [33], [35].

### Feature space of ORF

As mentioned above, there are 64 codon usage frequency features, 4148 Gene Ontology (GO) enrichment score features, 132 biochemical and physicochemical features, 24start codon features and 10 other features. The total featuresused in this study to represent an ORF sample would be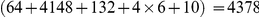.

### mRMR method

In this study, we used the Maximum Relevance and Minimum Redundancy (mRMR) feature selection method [36], [37] to rank 4378 features of each ORF considering both their relevance to translation rates and the redundancy among them. The mRMR selected features have maximum relevance with the translation rates and meanwhile minimally redundant, i.e., maximally dissimilar to each other. Both relevance and redundancy are measured with mutual information (MI), which is defined as follows:
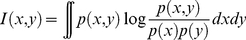
(2)where 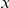 and 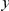 are two vectors, 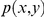 is the joint probabilistic density, 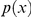 and 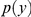 are the marginal probabilistic densities.

Let 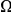 denotes the whole vector set containing all 4378 features, 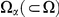 denotes the selected feature set with 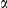 feature vectors, and 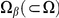 denotes the to-be-selected feature set with 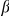 feature vectors. The relevance 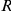 of a feature 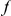 in 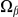 with the translation rate class 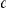 can be computed by equation (3):
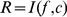
(3)


The redundancy 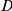 of a feature 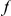 in 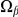 with all the features in 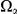 can be computed by equation (4):
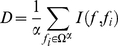
(4)


To select a feature 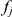 from 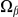 with maximum relevance with translation rates and minimum redundancy with selected features in 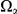, the mRMR function which integrates equation (3) and equation (4) is defined:
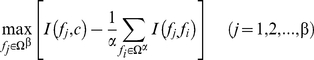
(5)


For a feature pool containing 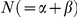 features, feature evaluation will be executed in 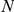
rounds. After these evaluations, a feature set 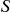 will be obtained:
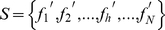
(6)where each feature has an mRMR order, representing at which round the feature is selected. The smaller order means more important.

### Nearest Neighbor Algorithm

To classify ORFs into different translation rate categories, the Nearest Neighbor Algorithm (NNA) was applied. Its basic idea is to predict a new ORF into its translation rate categories by comparing the features of this ORF with the features of those with known translation rate categories. The distance between two ORF vectors 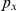 and 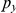 is defined as [30], [38]:
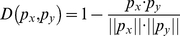
(7)where 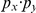 is the inner product of 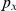 and 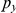, and 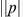 is the module of vector 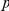. 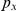 and 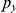 are consider to be more similar if 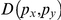 is smaller.

In NNA, an ORF with feature vector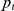 will be predicted as having the same translation rate class as its nearest neighbor which has the smallest 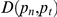. That is
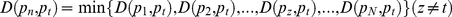
(8)where 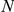 represents the number of training ORFs with known translation rates.

### Jackknife Cross-Validation Method

We used Jackknife Cross-Validation Method [38], [39], one of the most objective methods, to evaluate the performance of prediction. During Jackknife Cross-Validation, each ORF in the dataset is tested in turn by the translation rate predictor, which is trained by the other ORFs in the data set. Each ORF is involved in training 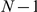 times and is tested exactly once. To evaluate the performance of the translation rate predictor, the prediction accuracy for the overall ORFs can be calculated as:
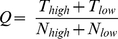
(9)where 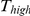 and 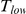 stand for the number of correctly predicted ORFs with high and low translation rate, respectively; 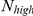
and 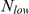 are the number of ORFs with high and low translation rate, respectively.

### Incremental Feature Selection (IFS)

When the mRMR step was completed, we obtained an ordered feature list but still do not know how many fore features in the list should be chosen. To determine the optimal number of features, Incremental Feature Selection (IFS) [30], [38] was applied by constructing 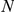
feature subsets of the feature list 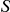 provided by mRMR. Starting from only the first feature 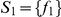, the feature subset 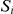 is defined as:
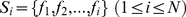
(10)by adding feature 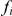 to the previous subset 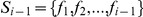.

For each feature subset 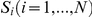, we calculated the prediction accuracy elevated by Jackknife Cross-Validation. The prediction accuracies with different feature numbers form an IFS curve with feature number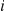 as its x-axis and the prediction accuracy as its y-axis.

### The correlation between features and translation rate

To identify the direction of the correlation between features selected by mRMR and IFS with translation rate, we calculated the point-biserial correlation coefficient between them. The point biserial correlation [40] is a measure of association between a continuous variable and a binary variable. Assume that X is the selected feature which is a continuous variable and Y is the translation rate which is binary. The point biserial correlation is calculated as
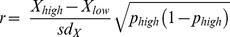
(11)where 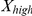 is the mean of 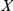 with high translation rate, 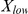 is the mean of 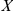 with low translation rate, 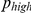 is the proportion of 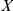 with high translation rate, 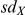 is the standard deviation of 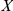. The point biserial correlation is positive when large values of 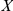
are associated with high translation rate and small values of 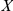
are associated with low translation rate.

## Results

### Identification of relevant features and construct translation rate prediction model

**Figure 1:**
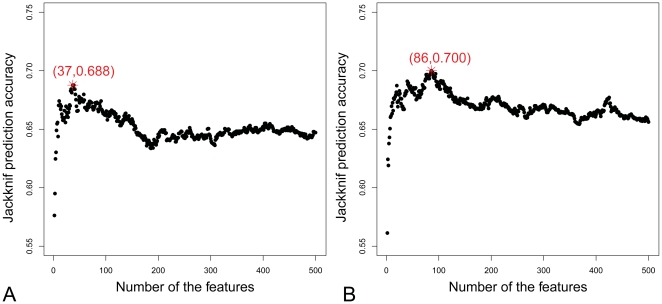
The IFS curves of translation rate prediction in rich and starvation condition. The IFS curves for (A) the translation rate prediction model of rich condition achieved the peak accuracy at 68.8% with 37 features and (B) the translation rate prediction model of starvation condition achieved the highest accuracy at 70.0% with 86 features.

Using mRMR method, we ranked and analyzed the top 500 relevant features to translation rate with Maximum Relevance Minimum Redundancy method. Each of them has the maximal relevance with translation rate and the minimal redundancy with other features. Then in Incremental Feature Selection (IFS) procedure, 500 prediction models were constructed using nearest neighbor algorithm with 1, 2, 3… 499 and 500 features respectively and tested by jackknife cross-validations as described above. The IFS results of rich and starvation condition were shown in **Figure 1** **(A)** and **Figure 1** **(B)**, respectively. It can be seen from **Figure 1** **(A)** that the translation rate prediction model of rich condition achieved the peak accuracy at 68.8% with 37 features. These 37 features formed the optimal feature set for translation rate prediction model of rich condition and are provided in **Table S1**. Similarly, in **Figure 1** **(B)**, the translation rate prediction model of starvation condition achieved the highest accuracy at 70.0% with 86 features. These 86 features formed the optimal feature set for translation rate prediction model of starvation condition and can be found in **Table S2**.

### Factors correlated with translation rate

**Figure 2:**
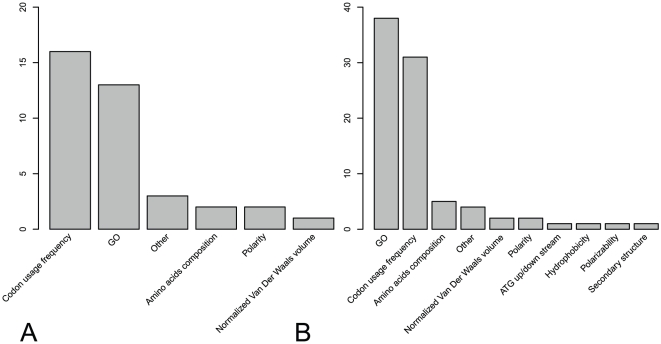
The numbers of each kind of features in optimal feature sets. The numbers of each kind of features for (A) the optimal 37-feature set of rich condition, (B) the optimal 86-feature set of starvation condition.

We compared the optimal 37-feature set of rich condition and the optimal 86-feature set of starvation condition and found there were 27 common features between them. These 27 common features are provided in **Table 2**. To identify what kinds of features are important for translation rate prediction, we calculated the numbers of each kind of features in the optimal feature set. **Figure 2** shows the numbers of each kind of features in (A) the optimal 37-feature set of rich condition, (B) the optimal 86-feature set of starvation condition. As we can see from **Figure 2**, **Table S1**, **Table S2** and **Table 2**, the following kinds of features are correlated with translation rate: (1) Codon usage frequency, (2) some Gene Ontology (GO) enrichment scores, (3) protein features (such as amino acids composition, polarity, normalized Van Der Waals volume) and (4) other features (such as Number of RNA binding proteins known to bind its mRNA product, Coding sequence length, Protein abundance and 5′UTR free energy).

**Table 2:** The common features for translation rate prediction in both rich condition and starvation condition.

Name	Feature Type	Point-Biserial Correlation (rich)	Point-Biserial Correlation (starvation)
ATA	Codon usage frequency	−0.3641809	−0.320724134
V123	Amino acids composition	0.217345654	0.249518281
CGA	Codon usage frequency	−0.297473206	−0.244839127
TCC	Codon usage frequency	0.251689274	0.234058044
NoofRNABindingProteins	Other (Number of RNA binding proteins known to bind its mRNA product)	0.22353164	0.194339726
GCT	Codon usage frequency	0.279887045	0.266483213
V126	Amino acids composition	−0.180096048	−0.149802124
GGA	Codon usage frequency	−0.208428434	−0.176300373
cds.length	Other (Coding sequence length)	0.097429773	−0.03025402
V72	Polarity	0.279590151	0.307614177
CGG	Codon usage frequency	−0.189139955	−0.147269889
PA	Other (Protein abundance)	0.141561548	0.120850079
AGG	Codon usage frequency	−0.199042873	−0.154301709
CCA	Codon usage frequency	0.282776605	0.283726919
ACC	Codon usage frequency	0.24618065	0.230897941
TGC	Codon usage frequency	−0.220759013	−0.173512017
GO:0005737	GO (GO:0005737_cytoplasm)	0.242558032	0.206209243
GCC	Codon usage frequency	0.268835706	0.270918872
GTA	Codon usage frequency	−0.212847373	−0.20408338
GO:0042277	GO (GO:0042277_peptide binding)	0.137845496	0.139232871
CTT	Codon usage frequency	−0.203855194	−0.190162108
TCT	Codon usage frequency	0.194907502	0.185575651
TAT	Codon usage frequency	−0.188268811	−0.173452245
AAC	Codon usage frequency	0.143590251	0.176587498
GO:0006878	GO (GO:0006878_cellular copper ion homeostasis)	0.134957407	0.131972094
V55	Normalized Van Der Waals volume	−0.19022717	−0.191407228

## Discussion

In this study, we have developed a new computational method to predict the translation rate by integrating various sequence-derived features and functional features. In rigorous jackknife cross-validation test, the predictor can achieve an overall prediction accuracy of 68.8% and 70.0% in rich and starvation conditions, respectively. We concluded that the following features are correlated with translation rate: codon usage frequency, some GO enrichment scores, protein features (such as amino acids composition, polarity, normalized Van Der Waals volume), number of RNA binding proteins known to bind its mRNA product, coding sequence length, protein abundance, and 5′UTR free energy. The following elucidations on these features confirmed their informative and importance in understanding the translation rate and translation mechanism in large-scale.

### Codon usage frequency

It has been reported by several studies that codon bias is the major factor for translation efficiency [31], [41]. In this study, we analyzed the relationship between the codon usage frequencies of ORFs and their translation rate. Our analysis not only confirmed the strong correlation between codon usage frequencies and translation efficiency, but also showed that more usage of which codon will result in high translation efficiency. It was found that the ORFs with higher frequencies of the following codons (AAC, TCT, ACC, TCC, GCC, GCT, CCA) tend to have higher rate of protein synthesis; on the other hand, higher frequency of the codons (ATA, CGA, TGC, GTA, GGA, CTT, AGG, CGG, TAT) relates to lower translation efficiency.

### Gene Ontology (GO) enrichment scores

We also analyzed 4148 Gene Ontology (GO) enrichment score features based on the STRING network context [17]. Interestingly, our analysis indicates that ORFs with different functions or subcellular locations will have different translation rate. The translation differences among different function groups have been mentioned before [42]. According to our analysis, in starvation condition, ORFs with cellular response function tend to have higher translation rate probably to improve the survival in this extreme condition. In starvation, high translation rate correlated with GO groups related to ‘cellular response’ (e.g. GO:0034605 - cellular response to heat, GO:0009409 - response to cold, GO:0009266 - response to temperature stimulus). An interesting contrast is the fact that the GO groups ‘GO:0005737 – cytoplasm’ and ‘GO:0001950 - plasma membrane’ are enriched with genes with high translation rate while the GO group ‘GO:0005634 – nucleus’ is enriched with genes with low translation rate. A possible explanation for this result is that in starvation condition in order to survive proteins in membrane and cytoplasm over-express, and genes related to DNA duplication (replication in the nucleus) under-express.

### Protein features

In our study, the protein features such as amino acids composition, polarity, normalized Van Der Waals volume were correlated with translation rate. The correlation between amino acid composition and protein abundance level has been reported [43] and it is possible that the amino acid composition may influence translation. The reason for the importance of protein features in translation efficiency prediction maybe that these features are strongly related to its function. And the translation difference among different function groups was mentioned in Ghaemmaghami's work [42].

### Other features

There are additional features that are useful for translation rate prediction. ‘Number of RNA binding proteins known to bind its mRNA product’, ‘Coding sequence length’, ‘Protein abundance’ and ‘5′UTR free energy’ are examples of such features. Firstly, there are a number of RNA binding proteins known to influence mRNA translation rate by bind its mRNA. For instance, RNA-binding proteins HuR and PTB promote the translation of Hypoxia-Inducible Factor 1α [44]. Cytochrome c mRNA translation is controlled by TIA-1 and HuR [45]. Furthermore, the correlation between protein abundance and the level of gene expression has been intensively studied (mainly on yeast). It was suggested that the relatively weak correlation between protein and mRNA abundance is due to different rates of translation and protein degradation [46]. Here we found that the ORFs with higher protein abundance tend to have higher translation rate. Thus, it is possible that the relatively weak correlation between the mRNA levels and protein abundance can be partially explained by the fact that translation rate is an important determinant of protein abundance that can't be estimated from mRNA levels. The last factor is 5′UTR free energy. It supports that previous studies that suggested that base-pairing potentials analysis of 5′UTR in various prokaryotes indicated that 5′UTR free energy is important for translation initiation [47].

Taken together, these sequence-derived and functional features are significantly-related to mRNA translation. Therefore, our prediction model might become a high throughput tool for annotating the translation rate of mRNAs. As a preliminary predictor of translation rate, the current model can only give the high or low categories of translation rate. When more in-depth understanding of translation is accumulated, the regression model might be tried to construct a more practical predictor which can directly estimate the translation rate.

## Supporting Information

Table S1 The features for translation rate prediction in rich condition.
(XLS)

Table S2 The features for translation rate prediction in starvation condition.
(XLS)
